# A Novel Approach Using DNA-Repair–Deficient Chicken DT40 Cell Lines for Screening and Characterizing the Genotoxicity of Environmental Contaminants

**DOI:** 10.1289/ehp.0900842

**Published:** 2009-06-26

**Authors:** Kyunghee Ji, Toshiaki Kogame, Kyungho Choi, Xin Wang, Jinyoung Lee, Yoshihito Taniguchi, Shunichi Takeda

**Affiliations:** 1 School of Public Health, Seoul National University, Seoul, Korea; 2 Graduate School of Medicine, Kyoto University, Kyoto, Japan

**Keywords:** alternative test methods development, arsenic, DNA repair, genotoxicity, high-throughput testing, UV radiation

## Abstract

**Background:**

Many bacterial or mammalian cell-based test systems, such as the Ames test, chromosomal aberration assays, or gene mutation assays, are commonly used in developed countries to detect the genotoxicity of industrial chemicals. However, the specificity is generally limited and the sensitivity is not sufficiently high. In addition, most assays cannot provide information on mechanisms of genotoxicity of a given chemical.

**Objectives:**

We aimed to establish a sensitive and fast screening method that is also capable of characterizing mechanisms of genotoxicity.

**Methods:**

We developed a novel bioassay employing gene-disrupted clones of the chicken DT40 B-lymphocyte line, which are designed to be deficient in several specific DNA repair pathways. Genotoxic chemicals can delay cellular proliferation in DNA-repair–deficient clones more significantly than in wild-type cells by interfering with DNA replication, thereby inducing DNA damage. In addition, we verified the validity of this assay by analyzing the genotoxicity of γ-rays, ultraviolet (UV) light, and sodium metaarsenite (NaAsO_2_). We also characterized DNA lesions induced by NaAsO_2_.

**Results:**

Genotoxicity of given stressors was successfully screened based on a comparison of proliferation kinetics between wild-type and DNA-repair–deficient mutants in 48 hr. We also found that NaAsO_2_ apparently induces at least two types of damage: chromosomal breaks and UV photoproduct-like DNA lesions.

**Conclusion:**

This bioassay is a reliable and sensitive screening tool for environmental mutagens as well as for further characterizing the nature of detected genotoxicity.

Although growing numbers of chemicals are being introduced into commercial use each year, the mutagenic and carcinogenic potential of these compounds is poorly understood. Historically, the Ames test, a bacterial reverse-mutation bioassay, is used most commonly to evaluate the genotoxicity of chemicals (Ames et al. 1975). This *in vitro* test is based on the detection of histidine-independent revertants in *Salmonella* strains after exposure to potential mutagens (Maron and Ames 1983). However, the Ames test is not a suitable model for the mutagenesis of metazoan cells, and thus may yield false-positive and false-negative results (Ashby and Styles 1978). A number of *in vitro* mammalian cell-based genotoxicity assays have been developed, including test methods for identifying micronuclei and mutations of marker genes (Bonassi et al. 2007; Kirkland et al. 2006). These assays are associated with high false-positive rates, so the validity of the individual test methods remains a subject of intense investigation (Kirkland et al. 2006). Another problem of these bioassays is limited sensitivity, because they use DNA-repair–proficient cells, which can quickly eliminate induced DNA damage.

One of the novel approaches for detecting genotoxicity is to use the fact that replicative DNA polymerases, in order to achieve the greatest accuracy during DNA synthesis, have not adapted to replicating template DNA that is damaged (Friedberg et al. 2005). Thus, subtle chemical modifications of template DNA strands are recognized by replicative DNA polymerases, which results in the stalling of replication. To avoid such stalling, excision repair pathways eliminate DNA lesions from DNA template strands before DNA replication. Moreover, if replicative DNA polymerases are blocked at damaged template strands, cells use two DNA repair pathways—homologous recombination (HR) and translesion DNA synthesis—to release the replication blockage (Sonoda et al. 2006). Translesion DNA synthesis polymerases are error-prone and are responsible for spontaneous, as well as ultraviolet (UV)-induced, mutagenesis in yeast (Stelter and Ulrich 2003). Unless all chromosomal DNA is replicated, cells are unable to proceed with the next cycle, leading to cellular suicide. As a consequence, exposure to genotoxic compounds may delay cell cycle progression and thus prolong cell cycle duration. Importantly, cells deficient in their ability to repair DNA damage would exhibit greater cell cycle delay and greater toxicity in response to exposure to a genotoxic agent, compared with DNA-repair–deficient wild-type cells. We hypothesized that the genotoxicity of chemicals could be detected by simply monitoring for differences in cellular proliferation rates between wild-type cells and isogenic clones deficient in DNA repair. Furthermore, as different types of DNA damage are repaired by different DNA repair pathways, consideration of differential cytotoxicity as a function of which DNA repair pathway has been knocked out would provide insight into the type(s) of genotoxicity induced.

The chicken DT40 B-lymphocyte line appears to be ideal for reverse genetic study. First, because of effective gene targeting, isogenic mutant clones of all known DNA damage response pathways are available for this cell line (Buerstedde and Takeda 1991). Second, the phenotype and karyotype of the cells is very stable. Third, the cells are maintained in suspension culture and are easy to culture. In addition, DT40 cells are commercially available (CRL-2111; American Type Culture Collection, Manassas, VA, USA). There are also critical advantages of DT40 cells over mammalian cell lines, in the identification of mutagenic agents by measuring cellular proliferation. First, approximately 70% of the cell cycle time is in the S phase, and the cells are unable to arrest at the G_1_/S boundary after DNA damage. Thus, DNA-damaging agents interfere with replicative DNA polymerases more effectively in DT40 cells than in mammalian cell lines. Second, the cell cycle duration is shorter than that of any mammalian cell lines; DT40 cells are capable of dividing up to three times per day (Hori et al. 2008; Zhao et al. 2007). Therefore, isogenic DNA repair mutants of DT40 cells provide a unique opportunity for screening various types of chemicals for genotoxicity by simply comparing rates of cell proliferation (or the extent of cytotoxicity) across different isogenic DNA repair mutants with that obtained for wild-type cells.

The purpose of the present study was to establish methods to screen for genotoxic chemicals at high throughput and to precisely characterize the type of damage induced by a genotoxic agent. To identify genotoxic agents using these DT40 clones, we used a colony-formation assay (CFA). However, this assay, which takes more than a week to complete, is not suitable for high-throughput screening, where 48 hr is the maximum duration for an experiment (Xia et al. 2008). We therefore evaluated the potential of a version of this assay based on investigating cellular proliferation over a shorter time period. To this end, we analyzed the cellular response of the isogenic DT40 mutants to γ-rays and UV light, which induce chromosomal breaks and UV photoproducts, respectively. We confirmed that in the new assay, monitoring cellular proliferation for only 2 days is sufficient to detect DNA damage induced by γ-rays and UV light. We also intended to test the utility of this novel method as a tool to precisely characterize the genotoxicity mechanism of given chemicals. To this end, we chose sodium meta-arsenite (NaAsO_2_) as a model chemical. Although arsenic is classified as a human carcinogen [International Agency for Research on Cancer (IARC]) 1987)], the Ames test cannot detect its mutagenicity (Abdullaev et al. 2001). We demonstrate here that our new protocol indeed works and that arsenic induces at least two types of DNA lesions: double-strand breaks (DSBs) and lesions that were repaired through the same pathway as UV-induced photoproducts.

## Materials and Methods

### Chemicals and cell cultures

NaAsO_2_ (98% purity), obtained from Sigma-Aldrich (St. Louis, MO, USA), was dissolved in distilled water for a 50-mM stock solution and stored at 4°C.

We used a panel of isogenic DT40 mutants, each defective in one of the major DNA damage pathways [e.g., DNA damage checkpoint, HR, nonhomologous end-joining, interstrand DNA cross-link repair, translesion DNA synthesis, nucleotide excision repair, and base excision repair [see Supplemental Material, Table 1, available online (doi:10.1289/ehp.0900842.S1 via http://dx.doi.org/)].

Cells (1 × 10^5^) were cultured in 100-mm Petri dishes with 10 mL RPMI 1640 medium (Nacalai Tesque, Kyoto, Japan) supplemented with 10% fetal calf serum (FCS; Equitech-Bio, Ingram, TX, USA), 1% chicken serum (Sigma, St. Louis, MO, USA), and 50 μM β-mercaptoethanol (Sigma) at 39.5°C in a humidified atmosphere of 5% CO_2_ and 95% air.

### Methylcellulose CFAs

We performed CFAs as described previously (Okada et al. 2002). Briefly, serially diluted cells were seeded into six-well plates. The cells were grown in 5 mL/well of 1.5% (wt/vol) methylcellulose (Aldrich, Milwaukee, WI, USA) containing 15% FCS, 1.5% chicken serum, and 50 μM β-mercaptoethanol at 39.5°C for 7–10 days. To test the response of the various clones to ionizing radiation, we plated cells into a medium containing methylcellulose; cells were incubated for 1 hr at 39.5°C and then irradiated with a ^137^Cs γ-ray source (GammaCell 40E; Nordion International, Kanata, Ontario, Canada) at doses ranging from 0 to 8 Gy. To test the various clones’ response to UV light, cells suspended in 0.5 mL phosphate- buffered saline (PBS) were introduced into six-well plates and irradiated with UV (254 nm wavelength) at doses ranging from 0 to 9 J/m^2^, followed by the addition of 5 mL of the complete medium containing methyl-cellulose. To understand the genotoxic mechanism of NaAsO_2_, we exposed various mutants to levels ranging from 100 μM to 2.4 mM and incubated them in methylcellulose containing the complete medium at 39.5°C for 7 days. At least three replicate colony determinations were carried out for each culture. The sensitivity of each mutant to γ-rays, UV, and NaAsO_2_ was assessed using D_10_ values (i.e., the dose that reduced the cell survival to 10%) (Nojima et al. 2005).

### Rapid survival assay

Cells (1 × 10^4^) were seeded into 12-well plates containing 3 mL/well of culture medium and incubated at 39.5°C for 48 hr. Cells were counted using flow cytometry as described by Takata et al. (1998). Briefly, the number of live cells was determined by mixing a 5-μg/mL propidium iodide (PI)–stained cell sample with a fixed number of 25-μm microspheres (Polysciences Inc., Warrington, PA, USA). Beads and viable cells were counted simultaneously as gated events, and cell numbers were calculated. Flow-cytometric analysis was performed on a FACScan (Becton Dickinson, Mountain View, CA, USA) using CellQuest software (Becton Dickinson). To test for differential sensitivity to γ-rays and UV light, cells were plated in culture medium, irradiated with ^137^Cs γ-rays or UV, and then incubated at 39.5°C for 48 hr. To evaluate the differential sensitivity to NaAsO_2_, cells were exposed to various concentrations of NaAsO_2_ (7.5–60 μM) and incubated at 39.5°C for 48 hr. At least three independent experiments were carried out to obtain individual data. We calculated the extent of cytotoxicity and D_10_ value in the same way used for the CFA.

### Measurement of adenosine-5′-triphosphate (ATP) in the rapid survival assay

Cells (1 × 10^3^) were seeded into 24-well plates containing 1 mL/well of culture medium and incubated at 39.5°C. ATP assays were carried out with 96-well plates using a CellTiter-Glo Luminescent Cell Viability Assay Kit (Promega Corp., Madison, WI, USA) at 24, 48, and 72 hr after chemical exposure. Briefly, we transferred 100 μL cell suspensions to the individual wells of 96-well plates, held the plates at room temperature for approximately 30 min, added 100 μL of CellTiter-Glo reagent, and mixed the contents for 2 min on an orbital shaker to induce cell lysis. The plate was then incubated at room temperature for 10 min to stabilize the luminescent signal. We measured luminescence using a Fluoroskan Ascent FL fluorometer (Thermo Fisher Scientific Inc., Waltham, MA, USA).

### Cell cycle analysis

Cells were seeded into 24-well plates containing PBS, exposed to 5 J/m^2^ UV, and then incubated in complete media for 6 hr. UV-irradiated cells and non-irradiated cells were labeled with 20 mM bromodeoxyuridine (BrdU; Amersham, Buckinghamshire, UK) for 10 min in 15-mL conical tubes, and then harvested and fixed overnight at 4°C with 70% ethanol. Cells were incubated first in 4N HCl, 0.5% Triton X-100 for 30 min at room temperature; second, in fluorescein isothiocyanate (FITC)–conjugated anti-BrdU antibody (Pharmingen, San Diego, CA, USA) for 1 hr at room temperature; and finally in 5 μg/mL PI in PBS. Between each incubation, cells were washed with PBS containing 2% FCS and 0.1% sodium azide. Flow cytometric analysis was then performed by FACScan using CellQuest software.

### Effect of a chemical scavenger on cellular viability

**Figure 1:**
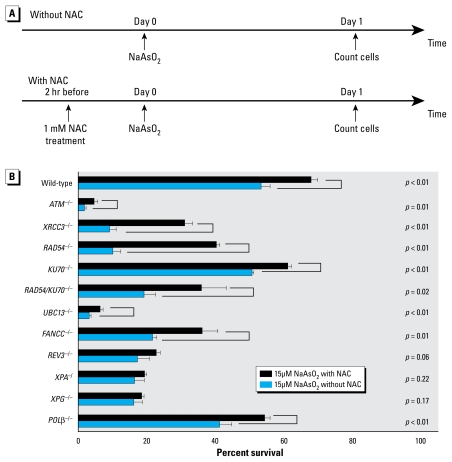
Protocol for treatment of cells deficient for DSB repair (*A*) and response of these cells to NaAsO_2_ with or without NAC (*B*).

To evaluate the involvement of reactive oxygen species (ROS) in DNA damage induction by NaAsO_2_, we pretreated DT40 cells with 1 mM *N*-acetyl-L-cysteine (NAC; Sigma), an ROS scavenger, 2 hr before NaAsO_2_ treatment (Figure 1A). Minimum concentration and treatment times were determined by preliminary experiments using 15 μM NaAsO_2_.

### Chromosomal aberration (CA) analysis

To prepare chromosome samples, we used a modification of the protocol described by Sonoda et al. (1998). Wild-type and mutant cells [*RAD54/KU70*^−/−^ and *XPA*^−/^ (*Xeroderma pigmentosum* complementation group A)] were treated with an RPMI 1640 medium containing 15 μM NaAsO_2_ and incubated at 39.5°C for 48 hr. To arrest cells at metaphase, we added 0.1 μg/mL colcemid (GIBCO-BRL, Grand Island, NY, USA) to the complete medium for the last 3 hr before harvest. Harvested cells were treated with 1 mL 75 mM KCl for 15 min at room temperature and fixed in 5 mL Carnoy’s solution (a freshly prepared 3:1 mixture of methanol and acetic acid). The cell suspension was dropped onto prewetted (50% ethanol) glass slides, flame dried, and then air-dried for 30 min. The slides were stained with a 3% Giemsa solution (Nacalai Tesque, Kyoto, Japan), pH 6.4 for 30 min and then rinsed with water and air-dried.

Analysis of chromosome structural aberrations was limited to the 11 autosomal macrochromosomes and the Z chromosome in Giemsa-stained metaphase cells (Sonoda et al. 1998). We observed 50 mitotic cells per culture for both wild-type and mutant cell types, and recorded the CAs (gaps and breaks) according to the International System for Human Cytogenetic Nomenclature (ISCN) system (Harnden and Klinger 1985). The ISCN defines a gap as a clear nonstaining region on a chromosome and a break as a discontinuity of a chromosome that shows a clear misalignment of the distal fragment of a broken chromosome. We used a magnification of 1,000× to score for CAs.

### Sister chromatid exchange analysis

For sister chromatid exchange (SCE) analysis, we modified the protocol described by Sonoda et al. (1999). Wild-type cells were cultured in the presence of 10 μM BrdU and 15 μM NaAsO_2_ for 16 hr (optimized for two cell cycles in preliminary experiments), with 0.1 μg/mL colcemid added for the last 2 hr. Harvested cells were treated with 75 mM KCl for 20 min and subsequently fixed with 5 mL Carnoy’s solution. Cells were fixed onto prewetted glass slides and dried on a 40°C plate. Dried slides were incubated with 10 μg/mL Hoechst 33258 in phosphate buffer (pH 6.8) for 30 min, and then rinsed with a McIlvaine solution [164 mM Na_2_HPO_4_, 16 mM citric acid (pH 7.0)]. Slides were irradiated with black light (λ = 352 nm) for 60 min and incubated in 2× SSC (1× SSC is 0.15 M NaCl plus 0.015 M sodium citrate) solution at 62°C for 1 hr before staining with a 3% Giemsa solution (pH 6.4) and subsequent microscopic analysis. We analyzed 11 auto-somal macrochromosomes and the Z chromosome from 50 mitotic cells per culture and recorded the frequency of SCEs. To score for SCE, we used a magnification of 1,000×.

### Statistical analysis

To analyze the data of the CFA, rapid survival assay (RSA), and ATP assay, we carried out a one-way analysis of variance (ANOVA) with Dunnett’s test. Normality and homogeneity of variances were verified using Shapiro–Wilk test and Levene’s test, respectively. We conducted an independent-samples *t*-test to compare treatments with and without NAC in SCE analysis. For all analyses, we used SPSS 15.0K for Windows (SPSS Inc., Chicago, IL, USA).

## Results

### Experimental design

In this study, we measured DNA damage responses of wild-type DT40 cells and isogenic DT40 mutants that were deficient in all major DNA repair pathways (Table 1). We did not include cells that were deficient in the mismatch repair pathway, because loss of this pathway increases cellular tolerance to DNA damage (Kawate et al. 1998). We tested the effect of γ-rays and UV light on cellular proliferation up to 3 days postirradiation by comparing their effect on colony formation. Pulse exposure of cells to γ-rays and UV light is a stringent test to evaluate the validity of a genotoxic assay, because continuous exposure of cells to DNA-damaging chemicals may augment more toxic effect on DNA repair mutant cells.

**Figure 2:**
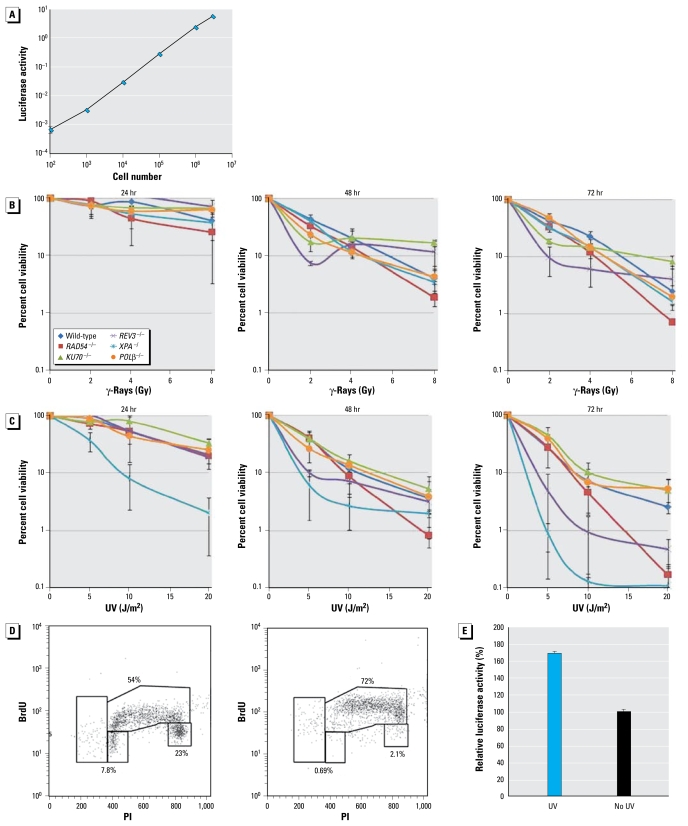
Analysis of cell number by measuring ATP in cellular lysates. (*A*) The relationship in the range from 10^2^/mL to 10^6^/mL DT40 cells with luciferase activity (the amount of ATP). (*B*, *C*) Luciferase activity 24, 48, and 72 hr after irradiation with γ-rays (*B*) and UV (*C*); the pattern of cellular proliferation was very similar between 48 and 72 hr after γ-irradiation (*B*). (*D*) The cell cycle 6 hr after exposure to 5 J/m^2^ UV (right) and with no UV irradiation (left) by BrdU pulse-labeling. (*E*) Amount of luciferase activity per cell. UV irradiation augmented the amount of ATP by 70% at 24 hr after UV compared with nonirradiated cells; 10^4^ cells were exposed to 5 J/m^2^ UV and subsequently incubated in 5 mL medium for 24 hr.

To evaluate the cellular response to DNA damage, we exposed wild-type and mutant cells (1 × 10^3^) to various doses of γ-rays and UV light and subsequently incubated them in 1 mL medium. Based on flow cytometric analysis, the number of live cells in each culture was determined 24, 48, and 72 hr after irradiation (Figure 2B,C). We then compared the results of this new protocol with those of a CFA, which is the most reliable standard assay for analyzing cellular response to DNA damage. We also indirectly assessed the number of living cells by measuring the level of ATP in cellular lysates, because this method is deemed more appropriate for high-throughput screening compared with flow cytometric analysis.

### Cell number at 48 hr after γ-ray and UV irradiation closely correlates with CFA data

**Figure 3:**
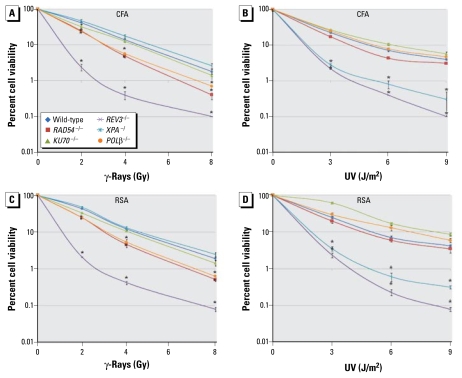
Sensitivity of repair-deficient DT40 mutant cells to γ-rays (*A,C*) and UV light (*B,D*) as shown by the CFA (*A,B*) and the RSA (*C,D*). Values shown are mean ± SD. *p < 0.05, compared with wild-type cells by one-way ANOVA.

Results for cellular sensitivity to γ-ray (Figure 3A,C) and UV irradiation (Figure 3B,D) for the indicated genotypes. Survival curves relative to the dose of irradiation were obtained from CFA (Figure 3A,B) and RSA (Figure 3C,D) 48 hr after irradiation. Table 1 shows the D_10_ vales of the indicated cells. For a quantitative comparison, we calculated the D_10_ values for the mutants relative to the D_10_ for wild-type cells, which was defined as 100%.

The two methods yielded very similar cellular survival data after γ- and UV irradiation. Data from both methods show the same order of γ-ray sensitivity among the mutant clones: cells deficient in *REV3*^−/−^ displayed the highest sensitivity to γ-rays, followed by *RAD54*^−/−^, *POL-*β^−/−^ (DNA polymerase-β), and *KU70*^−/−^ clones in descending order. The *XPA*^−/^ mutant showed no sensitivity. In cellular sensitivity to UV light, both methods consistently showed that the *REV3*^−/−^ and *XPA*^−/^ mutants exhibited the greatest sensitivity; *RAD54*^−/−^ mutants showed a mild sensitivity; and the *KU70*^−/−^ and *POL-*β^−/−^ mutants showed no elevated sensitivity. Moreover, in both measurements, the D_10_ values for γ-rays and UV light for wild-type cells were 4 Gy and 5 J/m^2^, respectively. Likewise, for γ-ray and UV irradiation D_10_ values were about four times smaller for the *REV3*^−/−^ and *XPA*^−/^ mutants than for wild-type cells. These observations indicate that the number of living cells at 48 hr after γ-rays and UV light closely correlates with the CFA data. Compared with pulse exposure to γ-rays and UV light, continuous exposure of cells to mutagenic chemicals may augment the toxic effect of those chemicals on DNA repair mutant cells. We therefore conclude that counting the number of living cells after a 48-hr exposure may be sufficient for reliable screening of genotoxic chemicals.

### Analysis of cell number by measuring ATP levels in cellular lysates

To estimate cell numbers, we evaluated ATP levels in cellular lysates because this method can be easily applied to robotic-based high-throughput screening. We first checked the relationship of the number of wild-type DT40 cells with the amount of ATP in cellular lysates and confirmed a linear relationship in the range from 10^2^/mL to 10^6^/mL DT40 cells (Figure 2A).

To test whether the ATP levels reliably represent the extent of cellular proliferation, we monitored the amount of ATP up to 72 hr postirradiation after γ-rays and UV light. The patterns of survival curves were very similar between 48 and 72 hr after irradiation (Figure 2B,C). Moreover, there is close correlation between the viability curves at 48 hr and data of methylcellulose CFA (Figure 3A,B). Thus, measurement of ATP in cellular lysates 48 hr after DNA damage may allow for accurate assessment of the number of DT40 cells in the screening of genotoxic chemicals.

We noticed that the patterns of the ATP-response curves substantially changed from the first to the second day after UV irradiation (Figure 2C). We hypothesized that UV irradiation may delay the progression of the cell cycle and thereby change the amount of ATP per cell at 24 hr after exposure. To address this hypothesis, we analyzed the cell cycle of wild-type cells 6 hr after 5 J/m^2^ UV by BrdU pulse-labeling (Figure 2D). As expected, UV irradiation increased the percentage of cells in the S phase, suggesting a delay in the progression of S phase due to replication stalling at large numbers of UV photoproducts on template strands (Edmunds et al. 2008). The resulting increase in the length of a single cell cycle might affect the amount of ATP per cell. We found that UV irradiation augmented the amount of ATP by 70% at 24 hr compared with nonirradiated cells (Figure 2E). Hence, the prolonged cell cycle arrest might account for no apparent increase in the UV sensitivity of *REV3*^−/−^ or *RAD54*^−/−^ clones at 24 hr after UV, although these clones are actually hypersensitive to UV.

### Analysis of cellular sensitivity of mutant clones to NaAsO_2_

**Figure 4:**
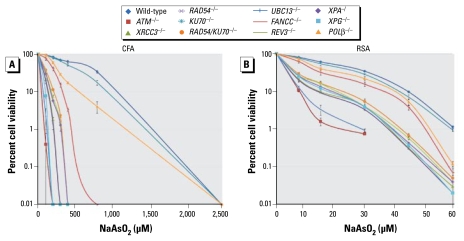
Toxicity profile of NaAsO_2_ for a panel of DT40 mutants, shown as the fraction of colonies arising from NaAsO_2_-treated cells obtained by CFA (*A*) and DT40 RSA (*B*). Values shown are mean ± SD.

To test the validity of our DT40-based assay for investigating the nature of DNA damage, we evaluated the sensitivity profile of DT40 mutant clones to NaAsO_2_ using the two methods: CFA and counting living cells (RSA) at 48 hr after continuous exposure to NaAsO_2_ (Figure 4). We normalized the sensitivity of the mutants (Table 2), as was performed for Table 1. As expected, both methods yielded similar sensitivity profiles of the DT40 mutant clones to NaAsO_2_. Surprisingly, the RSA (Figure 4B) seemed to detect the genotoxicity with a significantly higher sensitivity than did the CFA; the D_10_ arsenite concentration of wild-type cells was 1,400 μM in the CFA (Figure 4A) and only 45 μM in the RSA (Figure 4B).

### NaAsO_2_ induces DSBs during replication

Most cells defective in DNA repair displayed a hypersensitivity to NaAsO_2_ (Figure 1), suggesting that arsenite genotoxicity is suppressed by several DNA damage repair pathways. DT40 clones deficient in *RAD54* and *XRCC3* (X-ray repair complementing defective repair in Chinese hamster cells 3) displayed hypersensitivity, indicating that HR-mediated DSB repair contributes to cellular tolerance to arsenite. Cells deficient in *ATM* (ataxia telangiectasia-mutated), which plays a pivotal role in the response to a DSB (Mei et al. 2003), were also hypersensitive to arsenite. These observations suggest that arsenite may induce DSBs. To confirm this conclusion, we measured CAs in mitotic cells. As shown in Table 3, CAs were barely detectable in nonexposed cells, whereas both chromatid and chromosome-type breaks were frequently observed in cells exposed to arsenite. Thus, arsenite induces DSBs, which may lead to cell death. Although a defect in HR increased cellular sensitivity to NaAsO_2_, a defect in another major DSB repair pathway, nonhomologous end-joining (*KU70*^−/−^), had no impact on cellular sensitivity to arsenite. Likewise, *RAD54*^−/−^ and *RAD54*^−/−^*/KU70*^−/−^ clones displayed a comparable cellular tolerance to arsenite. These observations suggest that non-homologous end-joining is not necessarily involved in the repair of arsenite-induced chromosomal breaks, even in an HR-deficient background. Hence, arsenite-induced DSBs may arise mainly as a consequence of replication block in a sister chromatid, because this type of break is repaired exclusively by HR with another intact sister chromatid. The appearance of a chromosome-type break, where two sister chromatids are broken at the same site, might be attributable to failure of HR to complete DSB repair, leading to the occurrence of a DSB in the other intact sister chromatid during the condensation of chromosome towards the M phase (Sonoda et al. 2006).

**Figure 5:**
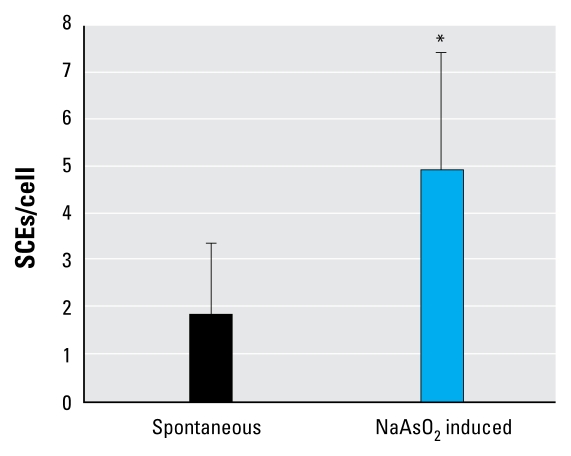
Frequency of SCEs (mean ± SD) in wild-type DT40 cells labeled with BrdU during two cell cycles with and without NaAsO_2_ treatment (15 μM) for 16 hr. See “Material and Methods” for additional details. *p < 0.05, compared with wild-type cells by independent-samples *t*-test.

To confirm the HR-mediated repair during DNA replication, we analyzed the frequency of SCEs (Figure 5), which reflects crossover-type HR associated with replication (Sonoda et al. 1999). The number of spontaneous SCEs in wild-type cells was 1.9 ± 1.5 per metaphase cell. Arsenite treatment increased the level of SCEs to 5.0 ± 2.5 per mitosis. This observation indicates that HR indeed plays a role in cellular response to arsenite during replication.

### NaAsO_2_ induces UV-damage-like DNA lesions

The sensitivity profile of the DNA repair mutants treated with NaAsO_2_ (Table 2) showed cellular responses very similar to those exposed to UV light (Table 1). In fact, cells deficient in nucleotide excision repair and HR exhibited a hypersensitivity to UV, whereas cells with a defect in nonhomologous end-joining or base excision repair did not. There are two possible explanations for this observation. First, arsenite induces base damage, which arrests replicative polymerases, leading to chromosomal breaks, as do UV-induced pyrimidine dimers (Friedberg et al. 2005). Thus, the primary base damage is eliminated by nucleotide excision repair, whereas the chromosomal breaks are repaired by HR. Second, arsenite may generate two different types of DNA damage, which are repaired separately by HR and nucleotide excision repair.

To address these two possibilities, we tested whether arsenite-induced DNA damage is mediated by ROS by pretreating cells with an antioxidant. Exposure to NAC itself had no effect on cellular proliferation (data not shown). Remarkably, pretreatment with NAC significantly reversed the cellular sensitivity to arsenite in the two HR mutants (*XRCC3*^−/−^ and *RAD54*^−/−^) (Table 4). In marked contrast, NAC treatment did not rescue the nucleotide excision repair mutants [*XPA*^−/^ and *XPG*^−/^ (*Xeroderma pigmentosum* complementation group G)] (Table 4). These results indicate that NaAsO_2_ may generate at least two distinctly different types of DNA damage: one mediated by ROS and the other independent of them. To confirm ROS-dependent DSBs, we measured CAs with and without NAC pretreatment. The NAC pretreatment significantly suppressed the level of CAs in the *RAD54*^−/−^/*KU70*^−/−^ mutant but had essentially no effect on the *XPA*^−/^ mutant (Table 3). These findings support the second possibility: NaAsO_2_ generates two different types of DNA damage, one induced by ROS leading to replication blocks and subsequent chromosomal breaks, and the other induced independently of ROS and eliminated by nucleotide excision repair, as are UV-induced photoproducts. Arsenite therefore may induce mutations by two distinct mechanisms. Chromosomal breaks cause chromosomal translocations as well as large deletions. Furthermore, because DT40 cells deficient in translesion synthesis polymerase (*REV3*^−/−^) cells were hypersensitive to arsenite, UV-photoproduct-like lesions may result in single-base substitutions through translesion synthesis. In summary, the sensitivity of isogenic-DNA-repair mutants to chemical compounds may be used to evaluate the type of induced DNA lesions and mechanisms underlying their mutagenesis.

## Discussion

In this study, we used chicken DT40 cells to develop an RSA suitable for the high-throughput screening of genotoxic environmental contaminants. This assay is also useful for the subsequent characterization of any identified genotoxicity. To screen for genotoxicity, we measured cellular proliferation during exposure of cells to chemical compounds, comparing isogenic clones deficient in individual DNA repair pathways with DNA-repair–proficient wild-type cells. This increases the sensitivity of the screening, because DNA repair mutants are not able to quickly remove induced DNA lesions. Moreover, the vast majority of the DNA-damaging agents interfere with DNA replication, and completion of replication is essential for cell cycle progression. In the RSA, DNA-repair–proficient wild-type cells serve as an important negative control to exclude positive data. We also identified, for the first time, the types of DNA lesions induced by arsenite.

The RSA proves to be an extremely sensitive tool for the detection of environmental mutagens for several reasons. Because DT40 cells are capable of proliferating faster than any mammalian cell line, larger numbers of cumulative cellular proliferation can be measured over a limited time for high-throughput screening. Moreover, induced DNA damage may more frequently interfere with DNA replication in the DT40 cells than in mammalian cells. This notion is suggested by the following two facts. First, the S phase constitutes about 70% of the whole cell cycle in DT40 cells, whereas most of the mammalian cell lines with a longer G_1_ phase are able to eliminate induced DNA damage before the cells enter the S phase. Second, induced DNA damage does not arrest the cell cycle at the G_1_/S boundary in DT40 cells, whereas mammalian cells may be arrested for as long as a few days at this boundary (Bao et al. 2001; Takao et al. 1999). Thus, if small numbers of induced DNA lesions are not quickly eliminated in DNA-repair–deficient DT40 cells, they may significantly reduce the growth kinetics of the cell. In contrast to DT40 cells, induced DNA damage may prevent mammalian cells from entering S phase for extended time in both wild-type cells and DNA-repair–deficient mutants without inducing apoptosis and thus decrease the sensitivity of the RSA. Hence, the use of DT40 cells is essential for the sensitive detection of environmental mutagens in the RSA. The assay is also appropriate for high-throughput screening.

Data obtained using chicken DT40 cells is likely to be relevant to mammalian cells because DNA damage-response pathways seem to be highly conserved in all eukaryotic cells. Furthermore, there is no apparent discordance in the phenotype if the ortholog genes are disrupted in mouse and DT40 cells. Indeed, phenotypic analysis of DNA-repair–deficient DT40 cells has contributed to the determination of previously unidentified genotoxicity, including that of the estrogen antagonist tamoxifen (Mizutani et al. 2004), nitric oxide (Wu et al. 2006), and formaldehyde (Ridpath et al. 2007). In addition, this bioassay can detect the genotoxicity of *N*-2-acetylaminofluorene and benzo[*a*]pyrene, which in mammalian cells are converted to toxic metabolic products only in hepatocytes (Okada et al. 2002). A U.S. National Institutes of Health (NIH) group (Sakamuru S, Huang R, Witt K, Austin CP, Tice RR, and Xia M, unpublished data) recently verified this conclusion, producing a sensitivity profile of DNA-repair DT40 mutants for 2,800 chemical compounds, some of which are known genotoxic agents. Because a number of DT40 mutants have been generated to analyze a variety of biochemical pathways, such as mitotic cell division, apoptosis, defense against oxidative stress, and cellular response to endoplasmic reticulum stress, the sensitivity profile of various DT40 mutants to test chemical compounds might allow for specific evaluation of the impact of the individual biochemical pathways that are selectively impaired in each mutant clone (Hori et al. 2008; Ruchand et al. 2006; Takada et al. 2009; Wang et al. 2006).

In the RSA, we counted cell number indirectly by measuring ATP concentrations in cell lysates. Similarly, the NIH group (Sakamuru S, Huang R, Witt K, Austin CP, Tice RR, and Xia M, unpublished data) needed to rely on the ATP assay, because the group could not directly count cell numbers in the high-throughput assay due to a technical limitation. Another limitation is that the high-throughput assay allows for incubating cells with test chemicals only for < 48 hr. Despite such experimental limitations, the NIH group successfully obtained highly reproducible data using the ATP assay. However, it remains elusive whether cellularity always linearly correlates with the amount of ATP. In the present study, we observed very good correlation between the amount of ATP and the number of the cells at the time later than 48 hr but not necessarily at 24 hr after exposure of genotoxic stress (data not shown). Future studies should examine whether non-genotoxic compounds (e.g., antimetabolites) significantly affect the level of ATP per cell, especially at early time points after exposure of cells to chemical compounds.

Arsenite is a naturally occurring metal and is widely distributed in the environment. The U.S. Environmental Protection Agency decreased the maximum contaminant level of arsenic in drinking water from 50 μg/L to 10 μg/L in 2001. In an *in vitro* study, Bau et al. (2002) indicated that arsenic generates oxidative DNA adducts and DNA–protein cross-links. In the present study, we showed that exposure of cells to 100 μM arsenite for 48 hr killed more DNA-repair–deficient cells than wild-type cells. We confirmed that arsenite induces at least two types of DNA damage: chromosomal breaks and lesions that were repaired through the same pathway as UV-induced photoproducts. We also showed a critical role of translesion DNA synthesis in cellular response to arsenite, demonstrating that arsenite can induce single-base substitutions through error-prone DNA synthesis during replication of damaged template strands. Recently, several studies have shown that methylation could increase the toxicity of the trivalent form of arsenic. According to Kligerman et al. (2003), methylated trivalent arsenicals are candidate ultimate genotoxic forms of arsenic, producing ROS that induce DNA strand breaks in human peripheral lymphocytes cells. Further studies are required to evaluate the methylated trivalent arsenicals in relation to arsenic toxicity using this proposed system.

In this article, we show that isogenic DT40 cell lines deficient in DNA damage responses provide a means for rapid and specific screening of genotoxicity and for evaluating their mechanism of action. The combination of our rapid screening assay and subsequent characterizations could be used to replace the current battery of bacterial and mammalian cell–based genotoxicity test methods. The high-throughput characterization of a large number of chemicals will be a valuable source of information for genotoxic chemical profiles, and ultimately contribute to the *in silico* prediction of genotoxicity from the structure of chemicals.

## Correction

In the manuscript originally published online, XPA^−/^ was inadvertently labeled XPA^−/−^ in Figures 1 and 3 and in “Materials and Methods” under “Chromosomal aberration (CA) analysis,” and XPG^−/−^ was labeled XPG^−/^ in Figure 3 and Table 2. These have been corrected here.

## Figures and Tables

**Table 1:** Relative γ-ray and UV irradiation D_10_ values of cells (mean ± SD), using methylcellulose CFA and RSA.

	γ-Ray		UV	
Cell type	CFA	RSA	CFA	RSA
Wild-type	100.00 ± 6.57	100.00 ± 2.91	100.00 ± 5.84	100.00 ± 4.49
*RAD54*^−/−^	65.99 ± 0.58*	68.50 ± 3.53*	83.39 ± 3.00	89.51 ± 5.77
*KU70*^−/−^	96.13 ± 4.35	93.77 ± 2.54	124.75 ± 2.74	165.17 ± 8.99
*REV3*^−/−^	24.92 ± 1.77*	25.64 ± 0.32*	33.72 ± 1.25*	34.83 ± 1.69*
*XPA*^−/^	111.95 ± 1.46	104.95 ± 4.50	36.05 ± 1.04*	38.95 ± 1.72*
*POL-*β^−/−^	67.51 ± 0.77*	71.25 ± 4.12*	104.49 ± 5.56	137.83 ± 12.11

* *p* < 0.05, compared with wild-type cells by one-way ANOVA.

**Table 2:** Relative NaAsO_2_ D_10_ value of cells (mean ±SD), using methylcellulose CFA and RSA.

Cell type	CFA	RSA
Wild-type	100.00 ± 1.23	100.00 ± 4.79
*ATM*^−/−^	3.34 ± 0.34*	17.46 ± 0.90*
*XRCC3*^−/−^	9.82 ± 0.90*	32.69 ± 2.72*
*RAD54*^−/−^	9.63 ± 0.68*	31.19 ± 2.02*
*KU70*^−/−^	85.07 ± 10.25*	93.58 ± 5.59
*RAD54*/*KU70*^−/−^	15.52 ± 0.90*	50.60 ± 5.43*
*UBC13*^−/−^	3.93 ± 0.90*	19.25 ± 1.79*
*FANCC*^−/−^	24.36 ± 0.90*	81.49 ± 6.06*
*REV3*^−/−^	13.56 ± 1.56*	45.52 ± 4.99*
*XPA*^−/^	12.77 ± 0.68*	44.93 ± 8.57*
*XPG*^−/−^	6.88 ± 0.90*	39.40 ± 4.10*
*POL-*β^−/−^	37.13 ± 1.77*	90.00 ± 5.61

Abbreviations: *ATM*, ataxia telangiectasia-mutated; *FANCC*, Fanconi anemia complementation group C; *UBC13*, ubiquit-in-conjugating enzyme 13; *XPG*, *Xeroderma pigmentosum* complementation group G; *XRCC3*, X-ray repair complementing defective repair in Chinese hamster cells 3.

* *p* < 0.05, compared with wild-type cells by one-way ANOVA.

**Table 3:** Frequency of CAs by genotype.

Cell	NaAsO_2_ (μM)	NAC (mM)	Chromatid breaks	Chromosome breaks	No. (%) of cells with chromosome aberrations
Wild-type	0	0	1	0	1 (2)
	0	1	1	0	1 (2)
	15	0	2	4	6 (12)
	15	1	2	1	3 (6)
*RAD54*/*KU70*^−/−^	0	0	2	0	2 (4)
	0	1	2	0	2 (4)
	15	0	3	9	12 (24)
	15	1	2	3	5 (10)
*XPA*^−/^	0	0	1	0	1 (2)
	0	1	1	0	1 (2)
	15	0	2	11	13 (26)
	15	1	2	10	12 (24)

Cells (*n* = 50) were treated with 15 μM NaAsO_2_ with or without NAC for 48 hr. No chromatid or chromosome gaps were observed.

**Table 4:** Relative D_10_ value (mean ± SD) of cells treated with 15 μM NaAsO_2_ with and without 1 mM NAC.

Cell	With 1 mM NAC	Without 1 mM NAC
Wild-type	53.37 ± 2.54	68.19 ± 1.64*
*ATM*^−/−^	1.81 ± 0.52	4.84 ± 0.79*
*XRCC3*^−/−^	9.21 ± 1.79	31.29 ± 2.09*
*RAD54*^−/−^	10.12 ± 2.19	40.37 ± 0.85*
*KU70*^−/−^	50.58 ± 0.56	61.27 ± 1.01*
*RAD54*/*KU70*^−/−^	19.09 ± 3.30	36.12 ± 7.05*
*UBC13*^−/−^	3.22 ± 0.50	6.49 ± 0.78*
*FANCC*^−/−^	21.78 ± 1.10	36.34 ± 4.39*
*REV3*^−/−^	17.26 ± 3.56	22.92 ± 0.89
*XPA*^−/^	16.55 ± 2.81	19.42 ± 0.39
*XPG*^−/^	16.15 ± 2.40	18.65 ± 0.74
*POL*-β^−/−^	41.08 ± 3.59	54.52 ± 1.47*

Abbreviations: *FANCC*, Fanconi anemia complementation group C; *UBC13*, ubiquitin-conjugating enzyme 13.

* *p* < 0.05, compared with wild-type cells by one-way ANOVA.
